# Crystal structure and Hirshfeld surface analysis of the fungicide metconazole

**DOI:** 10.1107/S205698902500310X

**Published:** 2025-04-08

**Authors:** Thaluru M. Mohan Kumar, Chaluvarangaiah Sowbhagya, Hemmige S. Yathirajan, Sean Parkin

**Affiliations:** a Department of Physical Sciences, Amrita School of Engineering, Amrita Vishwa Vidyapeetham, Bengaluru 560 035, India; b Department of Studies in Chemistry University of Mysore, Manasagangotri Mysuru 570 006 India; c Department of Chemistry University of Kentucky,Lexington KY 40506-0055 USA; Katholieke Universiteit Leuven, Belgium

**Keywords:** metconazole, fungicide, conazole, triazole, crystal structure, Hirshfeld surface analysis

## Abstract

Metconazole is a systemic triazole fungicide that inhibits the ergosterol bio­synthesis pathway. It is widely used in agriculture to control fungal infections, including rusts, fusarium and septoria diseases. The mol­ecular structure is a three-ring system, namely, 5-(4-chlorobenz­yl)-2,2-dimethyl-1-(1*H*-1,2,4-triazol-1-ylmeth­yl)cyclo­pentan-1-ol, C_17_H_22_ClN_3_O, consisting of a cyclo­pentan-1-ol with 1,2,4-triazol-1-ylmethyl, *gem*-dimethyl and 4-chloro­benzyl groups attached at the 1-, 2- and 5-positions of the cyclo­penta­nol ring. It has two stereocentres (cyclo­penta­nol positions 1 and 5) leading to four stereoisomers, with the (1*S*,5*R*) form being the most bioactive. Despite its agricultural significance, detailed crystallographic data remain scarce. This study reports the crystal structure and Hirshfeld surface analysis of racemic *cis*-metconazole [(1*S*,5*R*)/(1*R*,5*S*)], determined in the monoclinic space group *P*2_1_/*c* with two independent mol­ecules in the asymmetric unit (*Z*′ = 2). Both exhibit similar conformations, with minor differences in the cyclo­penta­nol ring puckering and the torsion angles between the three rings. The crystal packing consists of 2_1_-screw-related hy­dro­gen-bonded chains parallel to the *b* axis, with additional weak C—H⋯N and C—H⋯Cl contacts linking adjacent mol­ecules. Hirshfeld surface analysis indicates that inter­molecular inter­actions are dominated by contacts involving hy­dro­gen (96.1 and 96.7% for the two mol­ecules).

## Abstract 2

The crystal structure of [(1*S*,5*R*)/(1*R*,5*S*)]-*cis*-metconazole at 100 K is presented along with a Hirshfeld surface analysis com­paring the similarities of the atom–atom contacts involving the two independent mol­ecules in the asymmetric unit.

## 1. Chemical context

Metconazole is an agricultural fungicide discovered by the Kureha Corporation in 1986 (Kumazawa *et al.*, 2000 ▸). It is toxic to a broad range of fungal species (Ito *et al.*, 1999 ▸) by acting as a de­methyl­ation inhibitor (DMI) in the ergosterol biosynthesis pathway. It is used to control a range of fungal infections, including alternaria, rusts, fusarium and septoria diseases. Metconazole is also known to inhibit the synthesis of fungal cell membranes. As a systemic triazole fungicide, metconazole has been proposed for the control of Black Sigatoka disease (*Mycosphaerella fijiensis*) in bananas. Single and sequential applications of metconazole, alone or in combination with pyraclostrobin, to improve fusarium head blight control and wheat yield in Brazil were described by Spolti *et al.* (2013 ▸). Detailed applications of metconazole are well documented (Tateishi *et al.*, 2014 ▸). Enanti­oselective effects on photosynthetic activity in *Microcystis flosaquae* were reported by Li *et al.* (2021 ▸). Anti­fungal activities against the emerging wheat pathogen *Fusarium pseudogramine­arum* were recently published by Liu *et al.* (2023 ▸). A review of the pesticide risk assessment of metconazole was given by Álvarez *et al.* (2023 ▸), which suggested that it may cause liver damage in mammals. Recently, *in vitro* and *ex vivo* anti­fungal activities against the rice blast fungus *Pyricularia oryzae* have been reported (Fei & Hao, 2024 ▸). Stereoselective studies of metconazole in four types of fruit, including absolute configuration and SFC–MS/MS enanti­oseparation, degradation and risk assessment, were published by Diao *et al.* (2024 ▸).
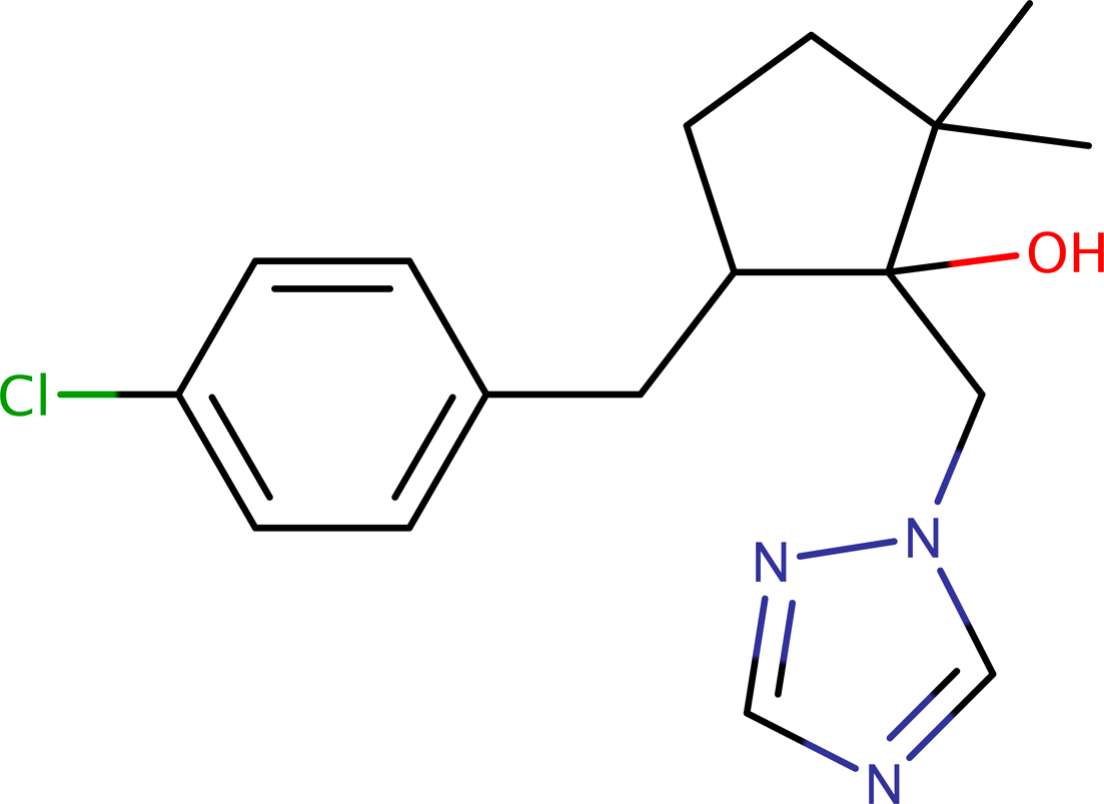


The structure of metconazole includes a cyclo­penta­nol ring substituted at the 1-, 2- and 5-positions by 1,2,4-triazol-1-ylmethyl, *gem*-dimethyl and 4-chloro­benzyl groups, respectively. It contains two chiral C atoms (C1 and C5), leading to four stereoisomers, *i.e.* two *cis* forms (1*S*,5*R*/1*R*,5*S*) and the two *trans* forms (1*S*,5*S*) and (1*R*,5*R*). The most bioactive is reported to be the (1*S*,5*R*) isomer (Ito *et al.*, 1999 ▸; He *et al.*, 2021 ▸). The crystal structure of the (1*S*,5*R*) isomer was reported by Ito *et al.* (1999 ▸), but the structure does not appear in either the Cambridge Structural Database (CSD, Version 5.46 of November 2024; Groom *et al.*, 2016 ▸) or the Crystallography Open Database (COD, accessed 23 March 2025; Gražulis *et al.*, 2009 ▸). In view of the agricultural importance and applications of metconazole, and the lack of readily accessible crystallographic structure details, this article reports the crystal structure and Hirshfeld surface analysis of a racemate of the *cis* forms, *i.e.* the (1*S*,5*R*/1*R*,5*S*) enanti­omorphs.

## 2. Structural commentary

**Figure 1:**
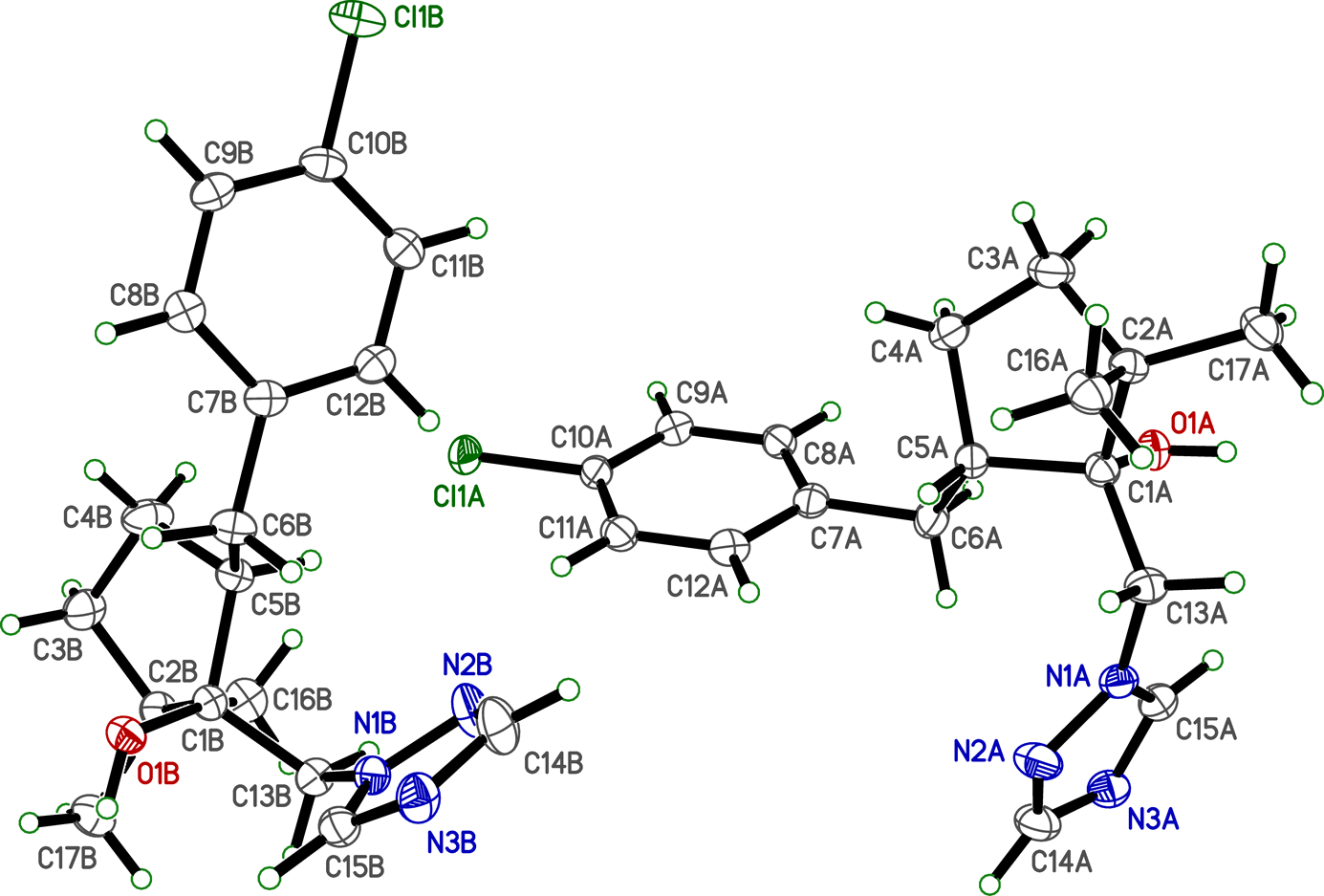
An ellipsoid plot (50% probability) of *cis*-metconazole. H atoms are drawn as small circles of fixed arbitrary radius.

**Figure 2:**
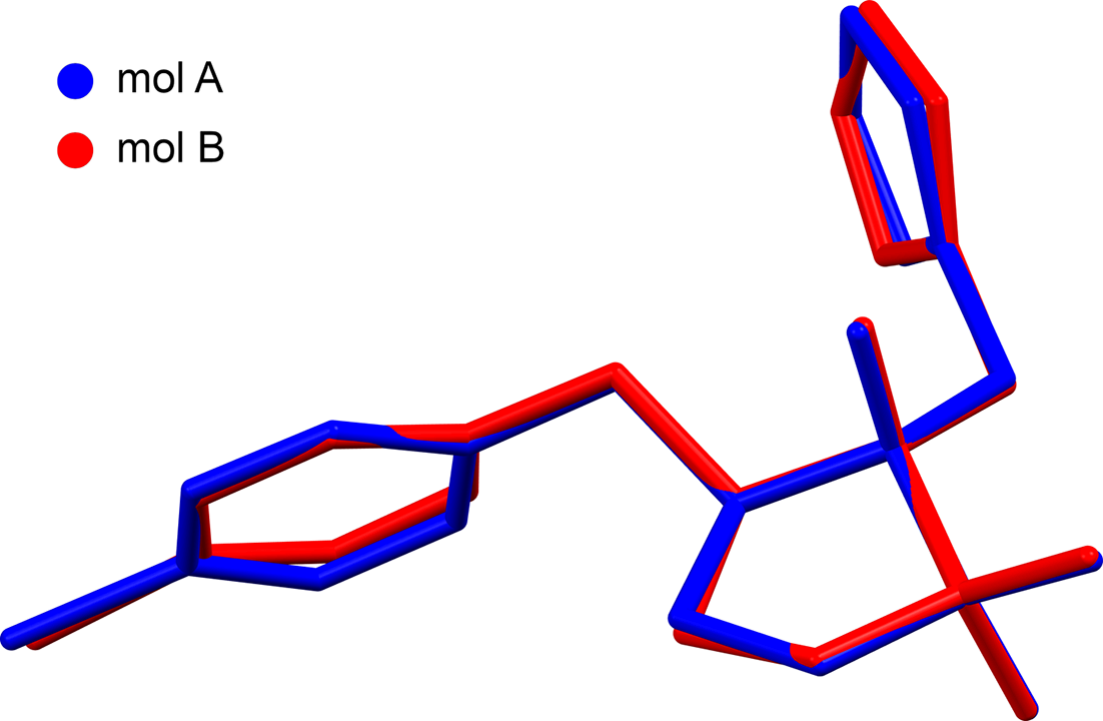
A least-squares overlay of the two symmetry-independent mol­ecules of *cis*-metconazole.

The structure of *cis*-metconazole presented here crystallizes in the monoclinic space group *P*2_1_/*c*, with two mol­ecules (*A* and *B* in Fig. 1 ▸) in the asymmetric unit (*Z*′ = 2). The structure is a three-ring system consisting of a central cyclo­penta­nol with 1,2,4-triazol-1-ylmethyl (and hydrox­yl) attached at C1, two methyl groups on C2 and a 4-chloro­benzyl group bonded to C5. Atoms C1 and C5 are stereogenic. In the assigned asymmetric unit, both mol­ecules are (1*R*,5*S*), but the crystallographic inversion requires that an equal amount of (1*S*,5*R*) must be present. The conformations of the two independent mol­ecules are broadly similar, as is evident in an overlay plot (r.m.s. deviation = 0.187 Å; Fig. 2 ▸). There are, however, minor differences. For example, in mol­ecule *A*, the maximum deviation from planarity of the cyclo­penta­nol ring is 0.2627 (10) Å at atom C1*A*, whereas for mol­ecule *B*, it is 0.2618 (11) Å at C2*B* owing to a slight change in ring pucker. The similarity in the conformations prompted us to check for a simpler structure with *Z*′ = 1 at room tem­per­a­ture, but none was found. The overall mol­ecular conformations are a consequence of rotation about the four rotatable bonds C5—C6, C6—C7, C1—C13 and C13—N1. For ease of com­parison, representative torsion angles qu­anti­fying the differences are presented in Table 1 ▸.

## 3. Supra­molecular features

**Figure 3:**
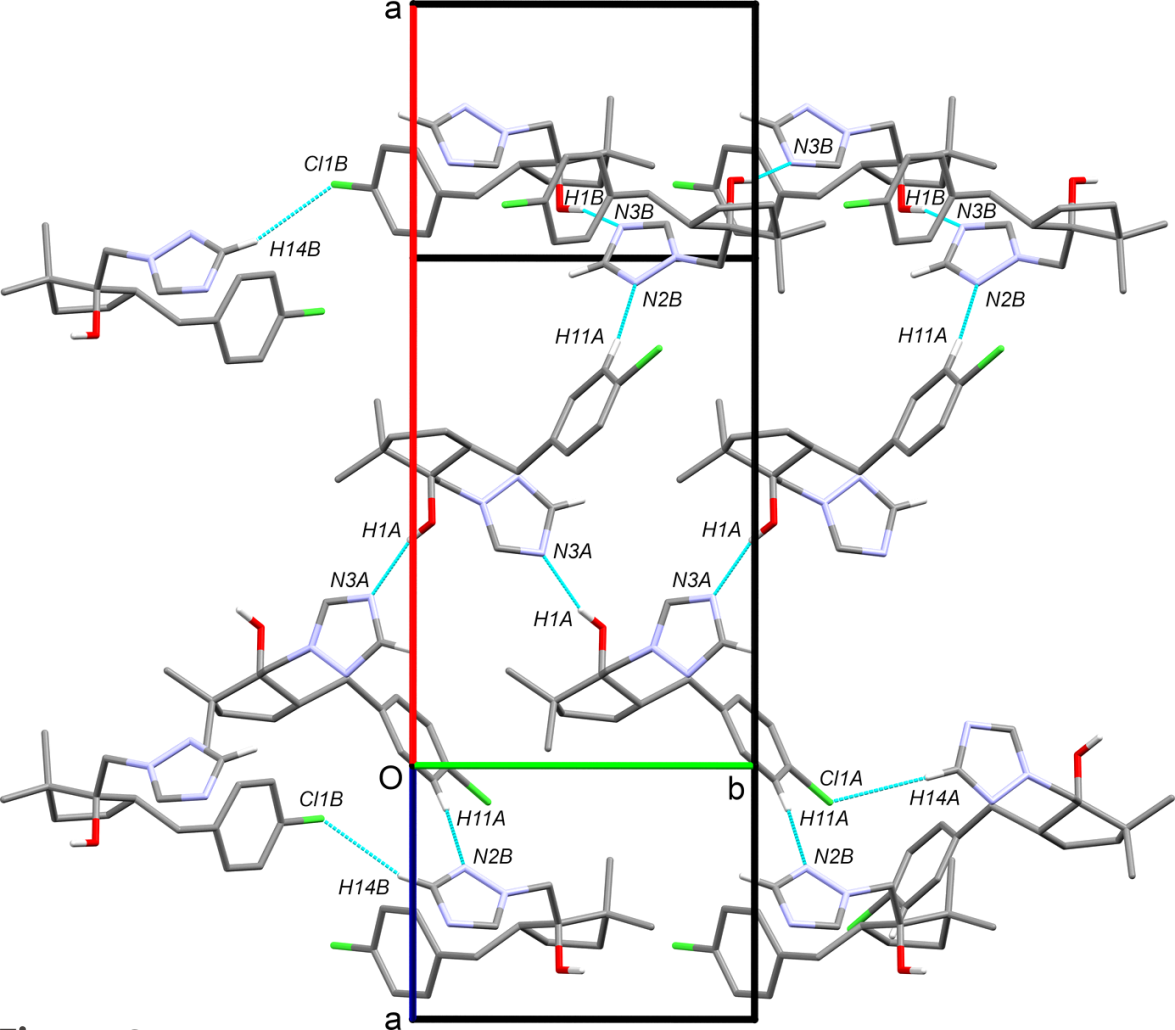
A partial packing plot, viewed down [103], showing O—H⋯N hy­dro­gen bonds, as well as C—H⋯N and C—H⋯Cl contacts, as dotted cyan lines.

There are only two conventional hy­dro­gen bonds in the crystal structure, namely, O1*A*—H1*A*⋯N3*A*^i^ [*D*⋯*A* = 2.9097 (18) Å] and O1*B*—H1*B*⋯N3*B*^iii^ [*D*⋯*A* = 2.8956 (18) Å] (the sym­metry codes are available in Table 2 ▸). These result in separate helical chains of 2_1_-screw-related *A* and *B* mol­ecules, each parallel to the *b* axis, as depicted in Fig. 3 ▸. The only noteworthy close contacts between the *A* and *B* mol­ecules are of the form C11*A*—H11*A*⋯N2*B* [*D*⋯*A* = 3.500 (2) Å]. The default suggestion for ‘potential hy­dro­gen bonds’ in *SHELXL* (Sheldrick, 2015*b* ▸) also flags weak contacts of the form C14*A*—H14*A*⋯Cl1*A*^ii^ [*D*⋯*A* = 3.9223 (18) Å] and C14*B*—H14*B*⋯Cl1*B*^iv^ [*D*⋯*A* = 3.6665 (18) Å] between *c*-glide-related mol­ecules. These are also shown in Fig. 3 ▸ and summarized in Table 2 ▸.

**Figure 4:**
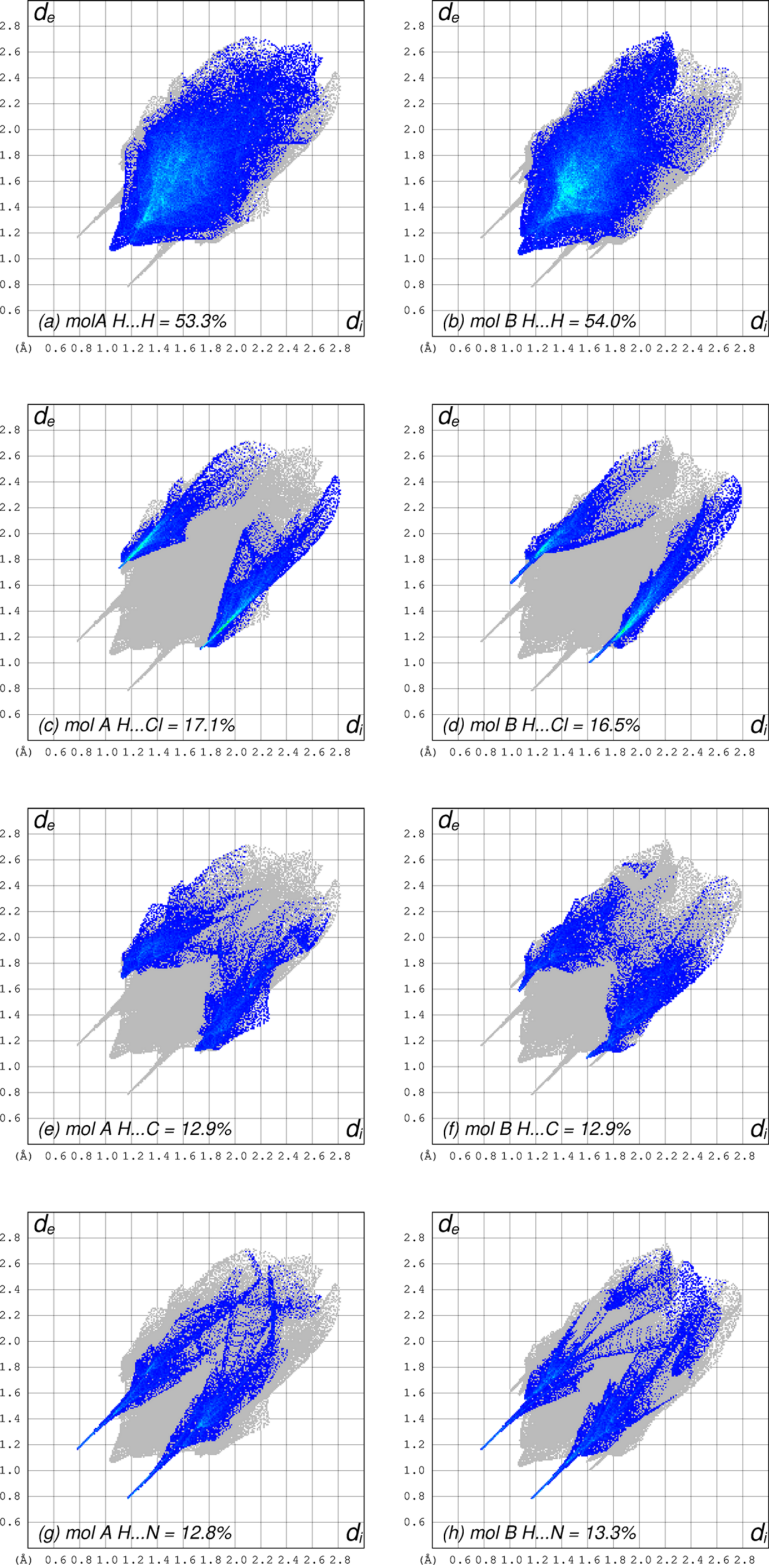
Hirshfeld surface two-dimensional fingerprint plots for each independent mol­ecule, arranged in pairs for mol­ecules *A* and *B*, respectively. (*a*)/(*b*) H⋯H, (*c*)/(*d*) H⋯Cl, (*e*)/(*f*) H⋯C and (*g*)/(*h*) H⋯N contacts. Reciprocal contacts are included in each case.

Separate Hirshfeld surface analyses of the two independent mol­ecules using *CrystalExplorer* (Spackman *et al.*, 2021 ▸) shows that both mol­ecules have very similar environments, with almost all atom–atom contacts (96.1% coverage for mol­ecule *A* and 96.7% for *B*) involving hy­dro­gen. These are depicted pairwise for H⋯H, H⋯Cl, H⋯C and H⋯N contacts in Fig. 4 ▸.

## 4. Database survey

A search of the Cambridge Structural Database (CSD, Version 5.46 of November 2024; Groom *et al.*, 2016 ▸) for the keyword ‘conazole’ returned 193 hits, while a search with both ‘conazole’ and ‘triazole’ produced 23 matches. A search using a mol­ecular fragment consisting of just the three-ring substructure gave two matches: an organic triazolium salt with [BF_4_]^−^ anions in which the heterocycle is fused to a substituted perhydro­penta­lene ring system (CSD refcode AWIGEV; Budny *et al.*, 2021 ▸) and a neutral com­pound (FEPHOA; Budny *et al.*, 2017 ▸), which differs from metconazole by the presence of an additional hydroxyl group at the 3-position of the cyclo­penta­nol ring. The crystal structure of the (1*S*,5*R*) isomer reported by Ito *et al.* (1999 ▸) was not found in either the CSD or the COD.

## 5. Synthesis and crystallization

The gift sample of metconazole was purified by column chromatography and recrystallized from methanol by slow evaporation to obtain X-ray-quality crystals (m.p. 386–389 K).

## 6. Data collection and refinement

None of the crystals were single, but appeared to be multiple domain two-com­ponent twins by reticular pseudomerohedry (*e.g.* Parkin, 2021 ▸). However, data images did not integrate well using two orientation matrices in the manner recommended by Sevvana *et al.* (2019 ▸). Nonetheless, it proved possible to excise most of the minor com­ponent from one specimen, so that the remaining minor twin fragment had a negligible effect on the measured diffraction maxima. This crystal was used for data collection. A second similarly treated crystal was later re-indexed at several tem­per­a­tures up to 294 K to check for any transition to a smaller *Z*′ = 1 structure, but no dramatic changes in unit-cell dimensions were observed.

All H atoms were found in difference Fourier maps. Carbon-bound H atoms were subsequently included in the refinement using riding models, with constrained distances set to 0.95 (C*sp*^2^—H), 0.98 (*R*CH_3_), 0.99 (*R*_2_CH_2_) and 1.00 Å (*R*_3_CH). Hydroxyl H atoms were also included using a riding model, but the O—H distances were refined. *U*_iso_(H) parameters were set to values of either 1.2*U*_eq_ or 1.5*U*_eq_ (*R*CH_3_ and OH) of the attached atom. Data collection and structure refinement statistics are summarized in Table 3 ▸.

## Supplementary Material

Crystal structure: contains datablock(s) I, global. DOI: 10.1107/S205698902500310X/vm2312sup1.cif

Structure factors: contains datablock(s) I. DOI: 10.1107/S205698902500310X/vm2312Isup2.hkl

Supporting information file. DOI: 10.1107/S205698902500310X/vm2312Isup3.cml

CCDC reference: 2441232

Additional supporting information:  crystallographic information; 3D view; checkCIF report

## Figures and Tables

**Table 1:** Conformation defining torsion angles (°) in metconazole

Torsion angle	Mol­ecule *A*	Mol­ecule *B*
C2—C5—C6—C7	−152.82 (19)	−151.88 (16)
C5—C6—C7—C8	113.52 (17)	116.02 (16)
O1—C1—C13—N1	−60.01 (17)	−56.18 (17)
C1—C13—N1—N2	−109.63 (16)	−102.44 (17)

**Table 2:** Hydrogen-bond geometry (Å, °)

*D*—H⋯*A*	*D*—H	H⋯*A*	*D*⋯*A*	*D*—H⋯*A*
O1*A*—H1*A*⋯N3*A*^i^	0.86	2.08	2.9097 (18)	164
C11*A*—H11*A*⋯N2*B*	0.95	2.62	3.500 (2)	155
C14*A*—H14*A*⋯Cl1*A*^ii^	0.95	2.98	3.9223 (18)	175
O1*B*—H1*B*⋯N3*B*^iii^	0.87	2.05	2.8956 (18)	163
C14*B*—H14*B*⋯Cl1*B*^iv^	0.95	2.75	3.6665 (18)	163

Symmetry codes: (i) 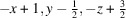; (ii) 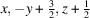; (iii) 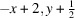, 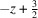; (iv) 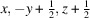.

**Table 3:** Experimental details

Crystal data	
Chemical formula	C_17_H_22_ClN_3_O
*M*_r_	319.82
Crystal system, space group	Monoclinic, *P*2_1_/*c*
Temperature (K)	100
*a*, *b*, *c* (Å)	21.1989 (6), 9.5096 (3), 17.6330 (5)
β (°)	110.483 (1)
*V* (Å^3^)	3329.95 (17)
*Z*	8
Radiation type	Mo *K*α
μ (mm^−1^)	0.24
Crystal size (mm)	0.22 × 0.20 × 0.11

Data collection	
Diffractometer	Bruker D8 Venture dual source
Absorption correction	Multi-scan (*SADABS*; Krause *et al.*, 2015 ▸)
*T*_min_, *T*_max_	0.903, 0.971
No. of measured, independent and observed [*I* > 2σ(*I*)] reflections	60403, 7671, 6719
*R*_int_	0.040
(sin θ/λ)_max_ (Å^−1^)	0.651

Refinement	
*R*[*F*^2^ > 2σ(*F*^2^)], *wR*(*F*^2^), *S*	0.038, 0.100, 1.15
No. of reflections	7671
No. of parameters	406
H-atom treatment	H atoms treated by a mixture of independent and constrained refinement
Δρ_max_, Δρ_min_ (e Å^−3^)	0.32, −0.24

Computer programs: *APEX5* (Bruker, 2023 ▸), *SHELXT* (Sheldrick, 2015*a* ▸), *SHELXL2019* (Sheldrick, 2015*b* ▸), *XP in *SHELXTL** (Sheldrick, 2008 ▸), *Mercury* (Macrae *et al.*, 2020 ▸), *SHELX* (Sheldrick, 2008 ▸) and *publCIF* (Westrip, 2010 ▸).

## supplementary crystallographic information

### 5-[(4-Chlorophenyl)methyl]-2,2-dimethyl-1-(1H-1,2,4-triazol-1-ylmethyl)cyclopentan-1-ol Crystal data

**Table:**

C_17_H_22_ClN_3_O	*F*(000) = 1360
*M**_r_* = 319.82	*D*_x_ = 1.276 Mg m^−^^3^
Monoclinic, *P*2_1_/*c*	Mo *K*α radiation, λ = 0.71073 Å
*a* = 21.1989 (6) Å	Cell parameters from 9892 reflections
*b* = 9.5096 (3) Å	θ = 2.8–27.5°
*c* = 17.6330 (5) Å	µ = 0.24 mm^−^^1^
β = 110.483 (1)°	*T* = 100 K
*V* = 3329.95 (17) Å^3^	Slab, colourless
*Z* = 8	0.22 × 0.20 × 0.11 mm

### 5-[(4-Chlorophenyl)methyl]-2,2-dimethyl-1-(1H-1,2,4-triazol-1-ylmethyl)cyclopentan-1-ol Data collection

**Table:**

Bruker D8 Venture dual source diffractometer	7671 independent reflections
Radiation source: microsource	6719 reflections with *I* > 2σ(*I*)
Detector resolution: 7.41 pixels mm^-1^	*R*_int_ = 0.040
φ and ω scans	θ_max_ = 27.6°, θ_min_ = 2.1°
Absorption correction: multi-scan (*SADABS*; Krause *et al.*, 2015)	*h* = −27→25
*T*_min_ = 0.903, *T*_max_ = 0.971	*k* = −12→12
60403 measured reflections	*l* = −22→22

### 5-[(4-Chlorophenyl)methyl]-2,2-dimethyl-1-(1H-1,2,4-triazol-1-ylmethyl)cyclopentan-1-ol Refinement

**Table:**

Refinement on *F*^2^	Secondary atom site location: difference Fourier map
Least-squares matrix: full	Hydrogen site location: difference Fourier map
*R*[*F*^2^ > 2σ(*F*^2^)] = 0.038	H atoms treated by a mixture of independent and constrained refinement
*wR*(*F*^2^) = 0.100	*w* = 1/[σ^2^(*F*_o_^2^) + (0.0277*P*)^2^ + 2.5387*P*] where *P* = (*F*_o_^2^ + 2*F*_c_^2^)/3
*S* = 1.15	(Δ/σ)_max_ = 0.001
7671 reflections	Δρ_max_ = 0.32 e Å^−^^3^
406 parameters	Δρ_min_ = −0.24 e Å^−^^3^
0 restraints	Extinction correction: *SHELXL2019* (Sheldrick, 2015b), Fc^*^=kFc[1+0.001xFc^2^λ^3^/sin(2θ)]^-1/4^
Primary atom site location: structure-invariant direct methods	Extinction coefficient: 0.0014 (3)

### 5-[(4-Chlorophenyl)methyl]-2,2-dimethyl-1-(1H-1,2,4-triazol-1-ylmethyl)cyclopentan-1-ol Special details

**Table:**

Experimental. The crystal was mounted using polyisobutene oil on the tip of a fine glass fibre, which was fastened in a copper mounting pin with electrical solder. It was placed directly into the cold gas stream of a liquid-nitrogen based cryostat (Hope, 1994; Parkin & Hope, 1998). Diffraction data were collected with the crystal at 100K.
Geometry. All esds (except the esd in the dihedral angle between two l.s. planes) are estimated using the full covariance matrix. The cell esds are taken into account individually in the estimation of esds in distances, angles and torsion angles; correlations between esds in cell parameters are only used when they are defined by crystal symmetry. An approximate (isotropic) treatment of cell esds is used for estimating esds involving l.s. planes.
Refinement. Refinement progress was checked using *PLATON* (Spek, 2020) and by an *R*-tensor (Parkin, 2000). The final model was further checked with the IUCr utility *checkCIF*.

### 5-[(4-Chlorophenyl)methyl]-2,2-dimethyl-1-(1H-1,2,4-triazol-1-ylmethyl)cyclopentan-1-ol Fractional atomic coordinates and isotropic or equivalent isotropic displacement parameters (Å^2^)

**Table:**

	*x*	*y*	*z*	*U*_iso_*/*U*_eq_	
Cl1A	0.68175 (2)	0.71920 (4)	0.40138 (2)	0.02457 (10)	
O1A	0.52795 (5)	0.05539 (12)	0.62641 (7)	0.0196 (2)	
H1A	0.5127 (4)	−0.008 (2)	0.6496 (11)	0.029*	
N1A	0.60358 (7)	0.20248 (14)	0.77571 (8)	0.0195 (3)	
N2A	0.64966 (7)	0.30544 (16)	0.80922 (9)	0.0277 (3)	
N3A	0.54519 (7)	0.37624 (15)	0.79895 (9)	0.0252 (3)	
C1A	0.59973 (7)	0.05243 (16)	0.65730 (9)	0.0172 (3)	
C2A	0.62713 (8)	−0.08738 (16)	0.63359 (10)	0.0197 (3)	
C3A	0.61181 (10)	−0.06495 (18)	0.54219 (10)	0.0268 (4)	
H3AA	0.645341	−0.114896	0.524671	0.032*	
H3AB	0.566442	−0.101316	0.510573	0.032*	
C4A	0.61530 (9)	0.09397 (18)	0.52911 (10)	0.0234 (3)	
H4AA	0.573718	0.126703	0.486173	0.028*	
H4AB	0.654268	0.116655	0.512683	0.028*	
C5A	0.62298 (8)	0.16560 (16)	0.61033 (9)	0.0185 (3)	
H5A	0.671963	0.183740	0.639758	0.022*	
C6A	0.58581 (9)	0.30658 (17)	0.59845 (10)	0.0230 (3)	
H6AA	0.536953	0.290105	0.571100	0.028*	
H6AB	0.592595	0.348816	0.652054	0.028*	
C7A	0.61005 (8)	0.40847 (16)	0.54868 (10)	0.0198 (3)	
C8A	0.56811 (8)	0.45113 (17)	0.47216 (10)	0.0207 (3)	
H8A	0.523805	0.413879	0.450445	0.025*	
C9A	0.58951 (8)	0.54694 (17)	0.42676 (10)	0.0213 (3)	
H9A	0.560076	0.575885	0.374879	0.026*	
C10A	0.65406 (8)	0.59929 (16)	0.45817 (9)	0.0196 (3)	
C11A	0.69756 (8)	0.55970 (17)	0.53428 (10)	0.0215 (3)	
H11A	0.741887	0.596966	0.555497	0.026*	
C12A	0.67496 (8)	0.46467 (17)	0.57866 (10)	0.0221 (3)	
H12A	0.704390	0.437069	0.630823	0.027*	
C13A	0.62454 (8)	0.07077 (17)	0.74931 (9)	0.0207 (3)	
H13A	0.607522	−0.008482	0.773112	0.025*	
H13B	0.674336	0.066043	0.770581	0.025*	
C14A	0.61194 (9)	0.40646 (19)	0.82161 (11)	0.0285 (4)	
H14A	0.630073	0.494312	0.844747	0.034*	
C15A	0.54260 (8)	0.24617 (18)	0.77086 (10)	0.0223 (3)	
H15A	0.502680	0.191605	0.750096	0.027*	
C16A	0.70333 (8)	−0.10486 (18)	0.67767 (11)	0.0249 (3)	
H16A	0.719344	−0.186311	0.655534	0.037*	
H16B	0.712545	−0.119355	0.735586	0.037*	
H16C	0.726673	−0.020057	0.670003	0.037*	
C17A	0.59222 (9)	−0.21937 (17)	0.64933 (11)	0.0254 (3)	
H17A	0.607908	−0.301802	0.627642	0.038*	
H17B	0.543382	−0.209774	0.622694	0.038*	
H17C	0.603015	−0.231026	0.707740	0.038*	
Cl1B	0.81854 (2)	0.26865 (4)	0.24588 (2)	0.02863 (11)	
O1B	0.96960 (5)	0.92837 (12)	0.60315 (7)	0.0201 (2)	
H1B	0.9829 (3)	0.993 (2)	0.6401 (13)	0.030*	
N1B	0.89595 (7)	0.76902 (14)	0.68325 (8)	0.0192 (3)	
N2B	0.85737 (7)	0.65081 (16)	0.66925 (9)	0.0288 (3)	
N3B	0.96104 (7)	0.60789 (15)	0.76048 (9)	0.0252 (3)	
C1B	0.89788 (7)	0.92607 (16)	0.57054 (9)	0.0170 (3)	
C2B	0.86934 (8)	1.06611 (16)	0.52487 (10)	0.0203 (3)	
C3B	0.88597 (9)	1.04776 (18)	0.44722 (10)	0.0261 (4)	
H3BA	0.856431	1.107926	0.403284	0.031*	
H3BB	0.933450	1.073313	0.457056	0.031*	
C4B	0.87378 (10)	0.89232 (19)	0.4246 (1)	0.0302 (4)	
H4BA	0.908484	0.856557	0.403812	0.036*	
H4BB	0.828929	0.879158	0.382263	0.036*	
C5B	0.87770 (8)	0.81331 (16)	0.50303 (9)	0.0192 (3)	
H5B	0.831491	0.779048	0.496496	0.023*	
C6B	0.92474 (8)	0.68569 (17)	0.51927 (10)	0.0218 (3)	
H6BA	0.970392	0.716866	0.523445	0.026*	
H6BB	0.927925	0.642304	0.571523	0.026*	
C7B	0.89944 (8)	0.57752 (16)	0.45240 (9)	0.0195 (3)	
C8B	0.93632 (8)	0.54384 (17)	0.40351 (10)	0.0213 (3)	
H8B	0.978630	0.587934	0.413145	0.026*	
C9B	0.91278 (8)	0.44719 (17)	0.34086 (10)	0.0220 (3)	
H9B	0.938695	0.424459	0.308174	0.026*	
C10B	0.85096 (8)	0.38482 (17)	0.32705 (9)	0.0211 (3)	
C11B	0.81342 (8)	0.41252 (17)	0.37572 (10)	0.0213 (3)	
H11B	0.771604	0.366651	0.366632	0.026*	
C12B	0.83852 (8)	0.50919 (17)	0.43823 (10)	0.0211 (3)	
H12B	0.813303	0.529015	0.472143	0.025*	
C13B	0.87017 (8)	0.89825 (17)	0.63853 (9)	0.0198 (3)	
H13C	0.881943	0.978517	0.676690	0.024*	
H13D	0.820441	0.892691	0.614957	0.024*	
C14B	0.89867 (9)	0.55898 (19)	0.71736 (11)	0.0308 (4)	
H14B	0.885667	0.464548	0.721624	0.037*	
C15B	0.95680 (8)	0.74128 (17)	0.73703 (9)	0.0214 (3)	
H15B	0.992286	0.807857	0.755978	0.026*	
C16B	0.79259 (8)	1.07684 (18)	0.50387 (11)	0.0264 (4)	
H16D	0.774938	1.154796	0.466017	0.040*	
H16E	0.782738	1.093744	0.553416	0.040*	
H16F	0.771280	0.988803	0.478829	0.040*	
C17B	0.90242 (9)	1.19719 (17)	0.57190 (11)	0.0267 (4)	
H17D	0.882680	1.281180	0.540294	0.040*	
H17E	0.950893	1.194861	0.581955	0.040*	
H17F	0.894934	1.199755	0.623668	0.040*	

### 5-[(4-Chlorophenyl)methyl]-2,2-dimethyl-1-(1H-1,2,4-triazol-1-ylmethyl)cyclopentan-1-ol Atomic displacement parameters (Å^2^)

**Table:**

	*U*^11^	*U*^22^	*U*^33^	*U*^12^	*U*^13^	*U*^23^
Cl1A	0.0309 (2)	0.01992 (19)	0.0269 (2)	−0.00292 (15)	0.01513 (16)	0.00167 (15)
O1A	0.0175 (5)	0.0193 (6)	0.0224 (5)	−0.0014 (4)	0.0075 (4)	0.0021 (4)
N1A	0.0222 (7)	0.0195 (7)	0.0175 (6)	0.0002 (5)	0.0076 (5)	−0.0022 (5)
N2A	0.0254 (7)	0.0280 (8)	0.0289 (8)	−0.0042 (6)	0.0086 (6)	−0.0110 (6)
N3A	0.0281 (7)	0.0237 (7)	0.0265 (7)	0.0016 (6)	0.0129 (6)	−0.0043 (6)
C1A	0.0165 (7)	0.0167 (7)	0.0187 (7)	−0.0001 (6)	0.0066 (6)	−0.0013 (6)
C2A	0.0224 (8)	0.0165 (7)	0.0214 (8)	−0.0004 (6)	0.0091 (6)	−0.0033 (6)
C3A	0.0369 (10)	0.0215 (8)	0.0222 (8)	0.0004 (7)	0.0106 (7)	−0.0044 (7)
C4A	0.0290 (9)	0.0237 (8)	0.0205 (8)	0.0002 (7)	0.0125 (7)	−0.0015 (6)
C5A	0.0203 (7)	0.0170 (7)	0.0199 (7)	−0.0012 (6)	0.0093 (6)	−0.0004 (6)
C6A	0.0276 (8)	0.0202 (8)	0.0262 (8)	0.0019 (6)	0.0155 (7)	0.0020 (6)
C7A	0.0234 (8)	0.0160 (7)	0.0231 (8)	0.0022 (6)	0.0121 (6)	−0.0009 (6)
C8A	0.0203 (7)	0.0201 (8)	0.0230 (8)	−0.0023 (6)	0.0092 (6)	−0.0033 (6)
C9A	0.0221 (8)	0.0227 (8)	0.0191 (7)	0.0019 (6)	0.0073 (6)	−0.0003 (6)
C10A	0.0249 (8)	0.0155 (7)	0.0217 (7)	−0.0010 (6)	0.0121 (6)	−0.0010 (6)
C11A	0.0198 (7)	0.0214 (8)	0.0234 (8)	−0.0019 (6)	0.0078 (6)	−0.0033 (6)
C12A	0.0240 (8)	0.0211 (8)	0.0201 (7)	0.0029 (6)	0.0064 (6)	0.0001 (6)
C13A	0.0230 (8)	0.0186 (8)	0.0203 (7)	0.0047 (6)	0.0072 (6)	−0.0009 (6)
C14A	0.0320 (9)	0.0247 (9)	0.0305 (9)	−0.0032 (7)	0.0133 (8)	−0.0100 (7)
C15A	0.0231 (8)	0.0234 (8)	0.0226 (8)	0.0006 (6)	0.0108 (6)	−0.0018 (6)
C16A	0.0231 (8)	0.0209 (8)	0.0322 (9)	0.0016 (6)	0.0117 (7)	−0.0035 (7)
C17A	0.0271 (8)	0.0175 (8)	0.0330 (9)	−0.0017 (6)	0.0124 (7)	−0.0030 (7)
Cl1B	0.0338 (2)	0.0224 (2)	0.0239 (2)	0.00359 (17)	0.00284 (16)	−0.00667 (16)
O1B	0.0182 (5)	0.0196 (6)	0.0208 (5)	−0.0008 (4)	0.0048 (4)	−0.0024 (4)
N1B	0.0215 (6)	0.0175 (6)	0.0188 (6)	−0.0008 (5)	0.0073 (5)	0.0014 (5)
N2B	0.0233 (7)	0.0231 (7)	0.0361 (8)	−0.0051 (6)	0.0055 (6)	0.0065 (6)
N3B	0.0261 (7)	0.0231 (7)	0.0251 (7)	0.0007 (6)	0.0074 (6)	0.0065 (6)
C1B	0.0169 (7)	0.0158 (7)	0.0180 (7)	0.0006 (6)	0.0057 (6)	0.0006 (6)
C2B	0.0235 (8)	0.0158 (7)	0.0209 (7)	0.0014 (6)	0.0070 (6)	0.0027 (6)
C3B	0.0363 (10)	0.0207 (8)	0.0224 (8)	0.0021 (7)	0.0117 (7)	0.0048 (7)
C4B	0.0478 (11)	0.0230 (9)	0.0185 (8)	0.0039 (8)	0.0102 (8)	0.0015 (7)
C5B	0.0208 (7)	0.0178 (7)	0.0180 (7)	−0.0002 (6)	0.0054 (6)	−0.0009 (6)
C6B	0.0235 (8)	0.0195 (8)	0.0199 (7)	0.0026 (6)	0.0043 (6)	−0.0029 (6)
C7B	0.0237 (8)	0.0165 (7)	0.0165 (7)	0.0037 (6)	0.0048 (6)	0.0014 (6)
C8B	0.0204 (7)	0.0208 (8)	0.0217 (8)	0.0013 (6)	0.0062 (6)	0.0016 (6)
C9B	0.0250 (8)	0.0224 (8)	0.0195 (7)	0.0053 (6)	0.0089 (6)	0.0006 (6)
C10B	0.0259 (8)	0.0161 (7)	0.0176 (7)	0.0040 (6)	0.0030 (6)	−0.0006 (6)
C11B	0.0216 (8)	0.0170 (7)	0.0233 (8)	0.0017 (6)	0.0055 (6)	0.0019 (6)
C12B	0.0236 (8)	0.0201 (8)	0.0209 (7)	0.0046 (6)	0.0093 (6)	0.0020 (6)
C13B	0.0215 (7)	0.0179 (7)	0.0201 (7)	0.0032 (6)	0.0075 (6)	0.0022 (6)
C14B	0.0296 (9)	0.0225 (9)	0.0369 (10)	−0.0032 (7)	0.0074 (8)	0.0092 (7)
C15B	0.0219 (8)	0.0220 (8)	0.0186 (7)	−0.0009 (6)	0.0051 (6)	0.0010 (6)
C16B	0.0253 (8)	0.0222 (8)	0.0288 (9)	0.0048 (7)	0.0059 (7)	0.0051 (7)
C17B	0.0321 (9)	0.0164 (8)	0.0285 (9)	0.0006 (7)	0.0068 (7)	0.0017 (7)

### 5-[(4-Chlorophenyl)methyl]-2,2-dimethyl-1-(1H-1,2,4-triazol-1-ylmethyl)cyclopentan-1-ol Geometric parameters (Å, º)

**Table:**

Cl1A—C10A	1.7490 (16)	Cl1B—C10B	1.7473 (16)
O1A—C1A	1.4259 (18)	O1B—C1B	1.4250 (18)
O1A—H1A	0.86 (2)	O1B—H1B	0.87 (2)
N1A—C15A	1.332 (2)	N1B—C15B	1.333 (2)
N1A—N2A	1.3631 (19)	N1B—N2B	1.3610 (19)
N1A—C13A	1.459 (2)	N1B—C13B	1.459 (2)
N2A—C14A	1.316 (2)	N2B—C14B	1.315 (2)
N3A—C15A	1.326 (2)	N3B—C15B	1.327 (2)
N3A—C14A	1.360 (2)	N3B—C14B	1.356 (2)
C1A—C13A	1.530 (2)	C1B—C13B	1.532 (2)
C1A—C5A	1.540 (2)	C1B—C5B	1.547 (2)
C1A—C2A	1.565 (2)	C1B—C2B	1.564 (2)
C2A—C17A	1.531 (2)	C2B—C17B	1.525 (2)
C2A—C16A	1.537 (2)	C2B—C3B	1.539 (2)
C2A—C3A	1.543 (2)	C2B—C16B	1.540 (2)
C3A—C4A	1.535 (2)	C3B—C4B	1.529 (2)
C3A—H3AA	0.9900	C3B—H3BA	0.9900
C3A—H3AB	0.9900	C3B—H3BB	0.9900
C4A—C5A	1.542 (2)	C4B—C5B	1.550 (2)
C4A—H4AA	0.9900	C4B—H4BA	0.9900
C4A—H4AB	0.9900	C4B—H4BB	0.9900
C5A—C6A	1.532 (2)	C5B—C6B	1.533 (2)
C5A—H5A	1.0000	C5B—H5B	1.0000
C6A—C7A	1.513 (2)	C6B—C7B	1.514 (2)
C6A—H6AA	0.9900	C6B—H6BA	0.9900
C6A—H6AB	0.9900	C6B—H6BB	0.9900
C7A—C8A	1.391 (2)	C7B—C12B	1.388 (2)
C7A—C12A	1.396 (2)	C7B—C8B	1.389 (2)
C8A—C9A	1.390 (2)	C8B—C9B	1.389 (2)
C8A—H8A	0.9500	C8B—H8B	0.9500
C9A—C10A	1.377 (2)	C9B—C10B	1.380 (2)
C9A—H9A	0.9500	C9B—H9B	0.9500
C10A—C11A	1.388 (2)	C10B—C11B	1.385 (2)
C11A—C12A	1.386 (2)	C11B—C12B	1.390 (2)
C11A—H11A	0.9500	C11B—H11B	0.9500
C12A—H12A	0.9500	C12B—H12B	0.9500
C13A—H13A	0.9900	C13B—H13C	0.9900
C13A—H13B	0.9900	C13B—H13D	0.9900
C14A—H14A	0.9500	C14B—H14B	0.9500
C15A—H15A	0.9500	C15B—H15B	0.9500
C16A—H16A	0.9800	C16B—H16D	0.9800
C16A—H16B	0.9800	C16B—H16E	0.9800
C16A—H16C	0.9800	C16B—H16F	0.9800
C17A—H17A	0.9800	C17B—H17D	0.9800
C17A—H17B	0.9800	C17B—H17E	0.9800
C17A—H17C	0.9800	C17B—H17F	0.9800
			
C1A—O1A—H1A	109.5	C1B—O1B—H1B	109.5
C15A—N1A—N2A	109.64 (13)	C15B—N1B—N2B	109.64 (13)
C15A—N1A—C13A	130.13 (14)	C15B—N1B—C13B	129.60 (14)
N2A—N1A—C13A	120.20 (13)	N2B—N1B—C13B	120.66 (13)
C14A—N2A—N1A	102.20 (14)	C14B—N2B—N1B	102.09 (14)
C15A—N3A—C14A	102.11 (14)	C15B—N3B—C14B	101.93 (14)
O1A—C1A—C13A	109.12 (12)	O1B—C1B—C13B	109.52 (12)
O1A—C1A—C5A	106.28 (12)	O1B—C1B—C5B	106.86 (12)
C13A—C1A—C5A	115.75 (13)	C13B—C1B—C5B	113.83 (13)
O1A—C1A—C2A	111.16 (12)	O1B—C1B—C2B	111.13 (12)
C13A—C1A—C2A	110.99 (12)	C13B—C1B—C2B	111.39 (12)
C5A—C1A—C2A	103.38 (12)	C5B—C1B—C2B	103.94 (12)
C17A—C2A—C16A	107.99 (13)	C17B—C2B—C3B	111.82 (14)
C17A—C2A—C3A	111.36 (14)	C17B—C2B—C16B	108.81 (14)
C16A—C2A—C3A	110.06 (13)	C3B—C2B—C16B	110.20 (14)
C17A—C2A—C1A	113.57 (13)	C17B—C2B—C1B	113.23 (13)
C16A—C2A—C1A	112.29 (13)	C3B—C2B—C1B	101.17 (12)
C3A—C2A—C1A	101.50 (12)	C16B—C2B—C1B	111.46 (13)
C4A—C3A—C2A	106.83 (13)	C4B—C3B—C2B	105.74 (13)
C4A—C3A—H3AA	110.4	C4B—C3B—H3BA	110.6
C2A—C3A—H3AA	110.4	C2B—C3B—H3BA	110.6
C4A—C3A—H3AB	110.4	C4B—C3B—H3BB	110.6
C2A—C3A—H3AB	110.4	C2B—C3B—H3BB	110.6
H3AA—C3A—H3AB	108.6	H3BA—C3B—H3BB	108.7
C3A—C4A—C5A	106.87 (13)	C3B—C4B—C5B	106.52 (13)
C3A—C4A—H4AA	110.3	C3B—C4B—H4BA	110.4
C5A—C4A—H4AA	110.3	C5B—C4B—H4BA	110.4
C3A—C4A—H4AB	110.3	C3B—C4B—H4BB	110.4
C5A—C4A—H4AB	110.3	C5B—C4B—H4BB	110.4
H4AA—C4A—H4AB	108.6	H4BA—C4B—H4BB	108.6
C6A—C5A—C1A	116.03 (12)	C6B—C5B—C1B	114.73 (13)
C6A—C5A—C4A	112.17 (13)	C6B—C5B—C4B	112.25 (13)
C1A—C5A—C4A	103.95 (12)	C1B—C5B—C4B	105.34 (13)
C6A—C5A—H5A	108.1	C6B—C5B—H5B	108.1
C1A—C5A—H5A	108.1	C1B—C5B—H5B	108.1
C4A—C5A—H5A	108.1	C4B—C5B—H5B	108.1
C7A—C6A—C5A	112.14 (13)	C7B—C6B—C5B	111.25 (13)
C7A—C6A—H6AA	109.2	C7B—C6B—H6BA	109.4
C5A—C6A—H6AA	109.2	C5B—C6B—H6BA	109.4
C7A—C6A—H6AB	109.2	C7B—C6B—H6BB	109.4
C5A—C6A—H6AB	109.2	C5B—C6B—H6BB	109.4
H6AA—C6A—H6AB	107.9	H6BA—C6B—H6BB	108.0
C8A—C7A—C12A	117.79 (15)	C12B—C7B—C8B	118.08 (15)
C8A—C7A—C6A	121.18 (15)	C12B—C7B—C6B	120.77 (14)
C12A—C7A—C6A	121.02 (15)	C8B—C7B—C6B	121.14 (15)
C9A—C8A—C7A	121.54 (15)	C9B—C8B—C7B	121.52 (15)
C9A—C8A—H8A	119.2	C9B—C8B—H8B	119.2
C7A—C8A—H8A	119.2	C7B—C8B—H8B	119.2
C10A—C9A—C8A	118.95 (15)	C10B—C9B—C8B	118.66 (15)
C10A—C9A—H9A	120.5	C10B—C9B—H9B	120.7
C8A—C9A—H9A	120.5	C8B—C9B—H9B	120.7
C9A—C10A—C11A	121.41 (15)	C9B—C10B—C11B	121.67 (15)
C9A—C10A—Cl1A	119.39 (12)	C9B—C10B—Cl1B	119.56 (12)
C11A—C10A—Cl1A	119.20 (12)	C11B—C10B—Cl1B	118.77 (13)
C12A—C11A—C10A	118.61 (15)	C10B—C11B—C12B	118.28 (15)
C12A—C11A—H11A	120.7	C10B—C11B—H11B	120.9
C10A—C11A—H11A	120.7	C12B—C11B—H11B	120.9
C11A—C12A—C7A	121.69 (15)	C7B—C12B—C11B	121.73 (15)
C11A—C12A—H12A	119.2	C7B—C12B—H12B	119.1
C7A—C12A—H12A	119.2	C11B—C12B—H12B	119.1
N1A—C13A—C1A	113.96 (13)	N1B—C13B—C1B	113.25 (13)
N1A—C13A—H13A	108.8	N1B—C13B—H13C	108.9
C1A—C13A—H13A	108.8	C1B—C13B—H13C	108.9
N1A—C13A—H13B	108.8	N1B—C13B—H13D	108.9
C1A—C13A—H13B	108.8	C1B—C13B—H13D	108.9
H13A—C13A—H13B	107.7	H13C—C13B—H13D	107.7
N2A—C14A—N3A	115.29 (15)	N2B—C14B—N3B	115.61 (16)
N2A—C14A—H14A	122.4	N2B—C14B—H14B	122.2
N3A—C14A—H14A	122.4	N3B—C14B—H14B	122.2
N3A—C15A—N1A	110.76 (15)	N3B—C15B—N1B	110.73 (15)
N3A—C15A—H15A	124.6	N3B—C15B—H15B	124.6
N1A—C15A—H15A	124.6	N1B—C15B—H15B	124.6
C2A—C16A—H16A	109.5	C2B—C16B—H16D	109.5
C2A—C16A—H16B	109.5	C2B—C16B—H16E	109.5
H16A—C16A—H16B	109.5	H16D—C16B—H16E	109.5
C2A—C16A—H16C	109.5	C2B—C16B—H16F	109.5
H16A—C16A—H16C	109.5	H16D—C16B—H16F	109.5
H16B—C16A—H16C	109.5	H16E—C16B—H16F	109.5
C2A—C17A—H17A	109.5	C2B—C17B—H17D	109.5
C2A—C17A—H17B	109.5	C2B—C17B—H17E	109.5
H17A—C17A—H17B	109.5	H17D—C17B—H17E	109.5
C2A—C17A—H17C	109.5	C2B—C17B—H17F	109.5
H17A—C17A—H17C	109.5	H17D—C17B—H17F	109.5
H17B—C17A—H17C	109.5	H17E—C17B—H17F	109.5
			
C15A—N1A—N2A—C14A	−0.61 (18)	C15B—N1B—N2B—C14B	0.57 (19)
C13A—N1A—N2A—C14A	177.59 (14)	C13B—N1B—N2B—C14B	177.23 (15)
O1A—C1A—C2A—C17A	−47.47 (17)	O1B—C1B—C2B—C17B	−45.87 (17)
C13A—C1A—C2A—C17A	74.17 (17)	C13B—C1B—C2B—C17B	76.54 (17)
C5A—C1A—C2A—C17A	−161.11 (13)	C5B—C1B—C2B—C17B	−160.47 (13)
O1A—C1A—C2A—C16A	−170.35 (13)	O1B—C1B—C2B—C3B	73.94 (15)
C13A—C1A—C2A—C16A	−48.71 (17)	C13B—C1B—C2B—C3B	−163.65 (13)
C5A—C1A—C2A—C16A	76.01 (15)	C5B—C1B—C2B—C3B	−40.67 (15)
O1A—C1A—C2A—C3A	72.15 (15)	O1B—C1B—C2B—C16B	−168.95 (13)
C13A—C1A—C2A—C3A	−166.21 (13)	C13B—C1B—C2B—C16B	−46.53 (17)
C5A—C1A—C2A—C3A	−41.49 (15)	C5B—C1B—C2B—C16B	76.45 (15)
C17A—C2A—C3A—C4A	151.19 (14)	C17B—C2B—C3B—C4B	159.06 (14)
C16A—C2A—C3A—C4A	−89.09 (16)	C16B—C2B—C3B—C4B	−79.78 (17)
C1A—C2A—C3A—C4A	30.01 (17)	C1B—C2B—C3B—C4B	38.25 (17)
C2A—C3A—C4A—C5A	−7.56 (18)	C2B—C3B—C4B—C5B	−21.50 (19)
O1A—C1A—C5A—C6A	43.87 (17)	O1B—C1B—C5B—C6B	34.43 (17)
C13A—C1A—C5A—C6A	−77.44 (17)	C13B—C1B—C5B—C6B	−86.60 (16)
C2A—C1A—C5A—C6A	161.00 (13)	C2B—C1B—C5B—C6B	152.03 (13)
O1A—C1A—C5A—C4A	−79.78 (14)	O1B—C1B—C5B—C4B	−89.54 (15)
C13A—C1A—C5A—C4A	158.91 (13)	C13B—C1B—C5B—C4B	149.44 (14)
C2A—C1A—C5A—C4A	37.35 (15)	C2B—C1B—C5B—C4B	28.07 (16)
C3A—C4A—C5A—C6A	−144.71 (14)	C3B—C4B—C5B—C6B	−129.92 (15)
C3A—C4A—C5A—C1A	−18.58 (17)	C3B—C4B—C5B—C1B	−4.40 (18)
C1A—C5A—C6A—C7A	−177.53 (13)	C1B—C5B—C6B—C7B	177.16 (13)
C4A—C5A—C6A—C7A	−58.26 (18)	C4B—C5B—C6B—C7B	−62.64 (18)
C5A—C6A—C7A—C8A	113.52 (17)	C5B—C6B—C7B—C12B	−63.93 (19)
C5A—C6A—C7A—C12A	−67.59 (19)	C5B—C6B—C7B—C8B	116.02 (16)
C12A—C7A—C8A—C9A	−0.3 (2)	C12B—C7B—C8B—C9B	1.5 (2)
C6A—C7A—C8A—C9A	178.62 (14)	C6B—C7B—C8B—C9B	−178.49 (15)
C7A—C8A—C9A—C10A	0.8 (2)	C7B—C8B—C9B—C10B	0.6 (2)
C8A—C9A—C10A—C11A	−0.8 (2)	C8B—C9B—C10B—C11B	−2.4 (2)
C8A—C9A—C10A—Cl1A	179.64 (12)	C8B—C9B—C10B—Cl1B	177.23 (12)
C9A—C10A—C11A—C12A	0.4 (2)	C9B—C10B—C11B—C12B	2.0 (2)
Cl1A—C10A—C11A—C12A	179.96 (12)	Cl1B—C10B—C11B—C12B	−177.58 (12)
C10A—C11A—C12A—C7A	0.0 (2)	C8B—C7B—C12B—C11B	−1.8 (2)
C8A—C7A—C12A—C11A	−0.1 (2)	C6B—C7B—C12B—C11B	178.13 (14)
C6A—C7A—C12A—C11A	−179.04 (15)	C10B—C11B—C12B—C7B	0.1 (2)
C15A—N1A—C13A—C1A	68.1 (2)	C15B—N1B—C13B—C1B	73.5 (2)
N2A—N1A—C13A—C1A	−109.63 (16)	N2B—N1B—C13B—C1B	−102.44 (17)
O1A—C1A—C13A—N1A	−60.01 (17)	O1B—C1B—C13B—N1B	−56.18 (17)
C5A—C1A—C13A—N1A	59.77 (18)	C5B—C1B—C13B—N1B	63.35 (17)
C2A—C1A—C13A—N1A	177.17 (12)	C2B—C1B—C13B—N1B	−179.52 (12)
N1A—N2A—C14A—N3A	0.3 (2)	N1B—N2B—C14B—N3B	−0.8 (2)
C15A—N3A—C14A—N2A	0.2 (2)	C15B—N3B—C14B—N2B	0.7 (2)
C14A—N3A—C15A—N1A	−0.59 (18)	C14B—N3B—C15B—N1B	−0.28 (18)
N2A—N1A—C15A—N3A	0.79 (19)	N2B—N1B—C15B—N3B	−0.19 (19)
C13A—N1A—C15A—N3A	−177.17 (15)	C13B—N1B—C15B—N3B	−176.45 (15)

### 5-[(4-Chlorophenyl)methyl]-2,2-dimethyl-1-(1H-1,2,4-triazol-1-ylmethyl)cyclopentan-1-ol Hydrogen-bond geometry (Å, º)

**Table:**

*D*—H···*A*	*D*—H	H···*A*	*D*···*A*	*D*—H···*A*
O1*A*—H1*A*···N3*A*^i^	0.86	2.08	2.9097 (18)	164
C11*A*—H11*A*···N2*B*	0.95	2.62	3.500 (2)	155
C14*A*—H14*A*···Cl1*A*^ii^	0.95	2.98	3.9223 (18)	175
O1*B*—H1*B*···N3*B*^iii^	0.87	2.05	2.8956 (18)	163
C14*B*—H14*B*···Cl1*B*^iv^	0.95	2.75	3.6665 (18)	163

Symmetry codes: (i) −*x*+1, *y*−1/2, −*z*+3/2; (ii) *x*, −*y*+3/2, *z*+1/2; (iii) −*x*+2, *y*+1/2, −*z*+3/2; (iv) *x*, −*y*+1/2, *z*+1/2.
